# Is Europe putting theory into practice? A qualitative study of the level of self-management support in chronic care management approaches

**DOI:** 10.1186/1472-6963-13-117

**Published:** 2013-03-26

**Authors:** Arianne Elissen, Ellen Nolte, Cécile Knai, Matthias Brunn, Karine Chevreul, Annalijn Conklin, Isabelle Durand-Zaleski, Antje Erler, Maria Flamm, Anne Frølich, Birgit Fullerton, Ramune Jacobsen, Zuleika Saz-Parkinson, Antonio Sarria-Santamera, Andreas Sönnichsen, Hubertus Vrijhoef

**Affiliations:** 1 Department of Health Services Research, CAPHRI School for Public Health and Primary Care, Maastricht University, Duboisdomein 30, PO Box 616, , Maastricht, 6200 MD, the Netherlands; 2 Health and Healthcare Research programme, RAND Europe, Cambridge, UK; 3 Faculty of Health Services Research and Policy, London School of Hygiene and Tropical Medicine, London, UK; 4 URC Eco Ile-de-France, Université Paris Est Créteil, Paris, France; 5 AP-HP Recherche Clinique Santé Publique, Hôpital Henri Mondor, Créteil, France; 6 MRC Epidemiology Unit, University of Cambridge, Cambridge, UK; 7 Institute of General Practice, Johann Wolfgang Goethe-University, Frankfurt, Germany; 8 Department for Evidence-based Medicine and Clinical Epidemiology, Danube University Krems, Krems, Austria; 9 Department of Health Services Research, Institute of Public Health, University of Copenhagen, Copenhagen, Denmark; 10 Institute of Preventive Medicine, Frederiksberg Hospital, Frederiksberg, Denmark; 11 Instituto de Salud Carlos III, Agencia de Evaluación de Tecnologías Sanitarias, Madrid, Spain; 12 Institute of General and Family Medicine, Witten/Herdecke University, Witten, Germany; 13 TRANZO Scientific Centre for Care and Welfare, Tilburg University, Tilburg, the Netherlands

**Keywords:** Chronic illness, Disease management, Self-management support, Comparative study, Qualitative research

## Abstract

**Background:**

Self-management support is a key component of effective chronic care management, yet in practice appears to be the least implemented and most challenging. This study explores whether and how self-management support is integrated into chronic care approaches in 13 European countries. In addition, it investigates the level of and barriers to implementation of support strategies in health care practice.

**Methods:**

We conducted a review among the 13 participating countries, based on a common data template informed by the Chronic Care Model. Key informants presented a sample of representative chronic care approaches and related self-management support strategies. The cross-country review was complemented by a Dutch case study of health professionals’ views on the implementation of self-management support in practice.

**Results:**

Self-management support for chronically ill patients remains relatively underdeveloped in Europe. Similarities between countries exist mostly in involved providers (nurses) and settings (primary care). Differences prevail in mode and format of support, and materials used. Support activities focus primarily on patients’ medical and behavioral management, and less on emotional management. According to Dutch providers, self-management support is not (yet) an integral part of daily practice; implementation is hampered by barriers related to, among others, funding, IT and medical culture.

**Conclusions:**

Although collaborative care for chronic conditions is becoming more important in European health systems, adequate self-management support for patients with chronic disease is far from accomplished in most countries. There is a need for better understanding of how we can encourage both patients and health care providers to engage in productive interactions in daily chronic care practice, which can improve health and social outcomes.

## Background

The rising incidence and prevalence of chronic conditions – especially cardiovascular disease, cancer, chronic respiratory illness, and diabetes – pose a threat to the long-term sustainability of health care delivery systems worldwide [1,2]. In many countries, the direct medical costs of managing chronic disease, and in particular multimorbidity, already take up a disproportionate share of the national health care budget [3-5]. Conversely, the quality of services provided to patients has remained largely sub-optimal, with consequences for disease control and patient experience [6,7].

In response, a wide range of innovative care concepts has been developed and implemented in many OECD countries [8,9]. One influential framework to conceptualize chronic care has been the Chronic Care Model (CCM) [10,11]. Conceived as an ‘evidence-based guide to comprehensive health care system redesign’ [12], it proposes six components to be core to providing high-quality, patient-centered chronic care: community resources and policies; the health care system; self-management support; decision support; delivery system design; and clinical information systems [10].

While the CCM recognizes the importance of interrelated change in multiple areas of care to better meet the needs of the chronically ill, self-management support has been identified as a key component of the framework [13]. Chronic illness confronts patients with a spectrum of needs that requires them to alter their behavior and engage in activities that promote physical and psychological well-being, which often have a more direct impact on disease control than the actions of health care professionals [14,15]. Evidence across multiple conditions suggests that effective self-management support can improve persons’ self-efficacy, i.e. their belief in their own ability to accomplish specific goals [16], and health-related behaviors, which, in turn, may impact their health and/or functional status [17-21]. Yet, in practice, approaches to self-management support appear to be the least implemented and most challenging area of chronic care management [22]. This is in part because self-management support will have to be targeted to meet individual needs, with consequent demands on providers’ time and resources in practice. Moreover, to help patients improve their self-efficacy requires communication skills and psychological counseling techniques which have not traditionally been part of most medical professionals’ training [23,24].

In this study, we review whether and how self-management support is integrated with existing approaches to chronic care management in 13 European countries, and the extent to which these approaches provide patients with the knowledge, skills, and confidence to effectively manage their condition. Nested within this review, we examine one country, the Netherlands, in more detail, assessing the level of and barriers to implementation of self-management support in current health care practice from the perspective of care professionals.

### Defining self-management support

Self-care and self-management are two distinct concepts that are often used interchangeably [25]. While self-care has been defined by the WHO as ‘the activities individuals, families, and communities undertake with the intention of enhancing health, preventing disease, limiting illness, and restoring health’ [26], self-management tends to refer to the active participation of patients in their treatment. [27] According to Corbin and Strauss [28], self-management concentrates on three distinct sets of activities: (1) medical management, which refers to tasks such as taking medication and adhering to dietary advice; (2) behavioral management, that is, learning new meaningful roles in the context of a specific condition; and (3) emotional management, which refers to dealing with the feelings of frustration, fright, and despair that are often experienced by chronically ill individuals. Self-management support is generally understood to target all three sets of tasks set out by the Corbin and Strauss framework. The CCM proposes that ‘by using a collaborative approach, providers and patients work together to identify problems, set priorities, establish goals, create treatment plans and solve issues along the way’ [29]. To facilitate patients to play such an active role in their care, patient education is usually a key part of self-management support [30]. Standardised interventions to support patients’ self-management furthermore may combine services available within health care (e.g. dietary advice, collaborative care planning) with services in the broader community (e.g. exercise programmes, peer support) [10,31].

## Methods

### Data template

This paper builds on work carried out within the DISMEVAL project (Developing and validating DISease Management EVALuation methods for European healthcare systems), a European collaborative project that aimed to identify ‘best practices’ in the area of disease management evaluation [32]. As part of DISMEVAL, a common template was developed for the collection of qualitative data on approaches to chronic care management in Europe. Template development was based on a structured questionnaire used in a previous study and informed, to great extent, by the CCM [8]. Thus, the template sought to gather information on: (1) the health system and policy context; and (2) the type and format of approaches to managing chronic disease, examining the nature and scope of the components identified by the CCM as crucial to effective chronic care management. The template was paper-based, written in English, and used simple checkboxes as well as open-ended questions. Where appropriate and relevant, sections included a glossary of definitions of terms and guidance for completion including examples and checklists. A shortened copy of the data collection template can be found elsewhere [32]. This paper reports the findings across countries pertaining to the CCM-component self-management support.

### Key informants

Data collection using the finalised template was undertaken by key informants in 13 countries, which were selected to capture the range of approaches to funding and governing healthcare, different levels of economic development, and geographical spread across Europe. We thus included: Austria, Denmark, England, Estonia, France, Germany, Hungary, Italy, Latvia, Lithuania, the Netherlands, Spain and Switzerland. Of these 13 countries, seven were represented by DISMEVAL project partners (Austria, Denmark, England, France, Germany, the Netherlands, and Spain) who were invited to complete the template. DISMEVAL project partners included two to four expert health service researchers per country. For countries not represented in DISMEVAL, key informants were identified from an established network of country experts in eight European countries (the International Healthcare Comparisons Network) [33]. Informants thus identified had to demonstrate expertise in the area of chronic disease and/or an understanding of the health policy and system context of the country in question as shown by relevant publications in the academic literature and/or roles in relevant government advisory bodies. One to four researchers and/or policymakers per country, who fulfilled these criteria, were selected as key informants for Estonia, Hungary, Italy, Latvia, Lithuania, and Switzerland (see Acknowledgments).

### Data collection

In completing the template, informants were asked to adopt an evidence-based, comprehensive approach, by making use of the best data available and cooperating with organisations involved in the management of chronic disease. Where appropriate and necessary, additional information was gathered through interviews with key stakeholders and reviews of work in progress, such as pilot projects and committee reports. As it was beyond the scope of DISMEVAL to provide a complete inventory of chronic care management approaches being implemented in the included countries, key informants were asked to present a sample of approaches considered representative of a given health system in terms of the type and setting of delivery model, providers involved, key strategies employed, and population covered. For each approach, respondents described whether and how self-management support activities were implemented, according to the CCM-related Assessment of Chronic Illness Care (ACIC) survey [34]; that is, patient education, collaborative care planning, provision of self-management tools, and structured follow-up. Principal data collection was carried out from June 2009 to December 2009, with sequential follow-up review untill July 2011 to complete missing data and clarify information.

### Case study

Template completion in the 13 countries was complemented by a case study of the Dutch DISMEVAL partner, which aimed to assess health professionals’ perspectives of the level of and barriers to implementation of self-management support activities in daily practice. Interviews were undertaken with a purposeful sample of 27 providers involved in disease management for type 2 diabetes in the Netherlands, using an ACIC-informed semi-structured interview guide [32]. Respondents represented an equal number of professionals from three different health care disciplines (i.e. managers, general practitioners (GPs), and nurses) and were selected from diverse care settings in terms of geographical location and practice size. The interviews were conducted mostly face-to-face, with five undertaken by telephone, by one member of the Dutch research team (AE) between February and June 2011. All interviews were audio recorded and transcribed.

### Data analysis

We used a general inductive approach to data analysis, in which emerging themes related to self-management support were identified through examination of the completed data template containing evidence from 13 countries. The data were analysed in detail by the lead author (AE) to identify key themes, which were discussed with and agreed by all coauthors. In total, three categories of themes were distinguished: (1) support mode and content (‘what’); (2) support format and materials (‘how’); and (3) support providers and locations (‘who and where’). Data were then organised into a purposely built matrix comprising the three emerged categories of themes, which facilitated systematic cross-country comparison of self-management support approaches in the 13 countries. For consistency, the same matrix was used to process and analyse the transcripts of the Dutch interviews concerning the implementation of self-management support in practice.

## Results

### An overview of self-management support in 13 countries

Additional file 1 provides an overview of approaches to chronic disease management or their equivalent in the 13 countries reviewed here. Keeping in mind that the overview is based on a sample of approaches considered representative of a given country context, the findings suggest that many countries have implemented a range of frequently small-scale chronic care management programmes at the local or regional level. In some cases, these have been conceptualized as pilot studies for subsequent roll-out to larger areas, while there are also examples of approaches that aim to target the entire population, in particular where these have been embedded within the existing primary care system. The majority of chronic care approaches in Europe as reviewed here involves some form of patient self-management support (see Additional file 1), although there are considerable differences in terms of: (1) mode and content (‘what’); (2) format and materials (‘how’); and (3) providers and locations (‘who and where’).

#### Support mode and content

Most chronic care approaches reviewed here involve education for self-management, frequently in the form of group-based exercises and/or one-to-one activities. For example, within the Austrian disease management programme ‘Therapie Aktiv’, nine hours of patient education including self-management training is offered in four modules with a group size of three to 12 patients. The German disease management programmes, which were introduced between 2003 and 2006 for six conditions (breast cancer, type 1 and 2 diabetes, coronary heart disease, asthma and chronic obstructive pulmonary disease), offer self-management support activities for each disease. Here too, self-management training is usually undertaken in groups, although individual support is an option.

With regard to content, the education offered within the reviewed support approaches tends to focus on three broad topics: (1) information about the disease; (2) information about healthy behavior (e.g. physical training sessions, nutritional consultation sessions, and smoking cessation programmes); and (3) practical instructions concerning, for instance, blood glucose monitoring, foot examination, or insulin injection.

Respondents from 10 countries – except Latvia, Lithuania and Spain – reported that patients are involved in setting care goals and developing individual treatment plans (i.e. collaborative care planning). Patients’ needs, activities, problems and accomplishments are regularly assessed by means of structured follow-up in all countries except for Latvia, where self-management support appears relatively most underdeveloped. Within the German disease management programmes, individual treatment goals (concerning, for example, blood pressure, weight, and exercise) are discussed between patients and their doctors during regular three to six-monthly follow-up consultations. French patients enrolled in provider networks have a ‘personal care plan’, which is set up jointly with their physician and contains treatment goals as well as concrete care measures.

#### Support format and materials

Respondents from all but two countries (Latvia and Lithuania) reported the use of support materials to help patients manage their chronic disease. In some programmes, the format of support is limited to written information, such as brochures detailing provisions for access to health promotion and disease prevention services. An example is the Delta Physician Network programme in Geneva, Switzerland. German statutory health insurance (SHI) funds provide disease management patients with information leaflets about their condition in situations where they develop complications, do not comply with treatment and referral guidelines, fail to reach their treatment goals (e.g. target blood pressure), or miss appointments for follow-up and patient training. In most of the reviewed chronic care programmes, such as the Danish SIKS project and the Italian Raffaello project, written information materials complement oral patient education, which offers patients an opportunity to ask questions and discuss problems with health professionals. In Austria, education is also provided through awareness campaigns and targeted lectures for stroke patients within ‘integrated-stroke-care-Upper-Austria’ and similar projects in other states.

Respondents from four countries reported that local projects offer patients access to interactive websites, such as ‘Gluco.net’ in Hungary, ‘DIEP.info’ in the Netherlands, and comparable initiatives in France and Switzerland. In Andalucía, Spain, a school for patients was developed in 2008 to instruct individuals on the management of their chronic illness. In addition, the region has a 24-hour health service telephone line which patients can contact in case of doubts or questions. Telephone-based support is also provided in projects in Germany, Hungary, and Italy, as well as in the French disease management programme for diabetes (the Sophia project) to provide patients with personalized information on how to manage their disease.

Respondents from three countries reported the use of peer support in specific chronic care programmes. In Switzerland, peer support is part of a regional breast cancer clinical pathway introduced between 2008 and 2009, which is hospital-based and targets adults with breast cancer. In Estonia, peer support is offered through patient associations. Some of the Partnerships for Older People Projects (POPP) in England also offer peer support, for example in the form of broader health and well-being advice from other older people.

#### Support providers and locations

In all reviewed countries, self-management support is offered by health professionals including physicians and/or, more often, trained nurses. The latter is the case in Austria, Denmark, England, Estonia, France, Germany, Hungary, Italy, the Netherlands, and Switzerland. In England, the 2004 NHS Improvement Plan introduced the concept of the ‘community matron’, a specialized senior nursing role undertaking intensive home-based case management for elderly people at risk of hospitalization and other high-intensity care users.

In Denmark, self-management training is provided either in outpatient clinics’ associated with the hospital, i.e. for patients with severe chronic disease, or in the municipalities for non-complex patients. In Estonia, support can be provided at home by a nurse or social worker. For some conditions, community services, such as the Estonian Parkinson’s Association, may be involved in supporting patients by providing information materials, organising lectures, and offering practical training and mentoring. Within the Italian Leonardo project, a care manager, usually a specialist nurse, guides patients in raising their level of self-awareness. Although few of the reviewed approaches to self-management support in the 13 countries use lay expertise, one well-known example is the English Expert Patient Programme, a six-week lay-led educational course for chronically ill patients.

### Case study: Dutch health professionals’ perspective on the implementation of self-management support in daily practice

The Dutch approach to structured disease management for chronic conditions and, in particular, the self-management support activities included in that approach can be summarized as follows. In January 2010, after several years of experiments, a bundled payment system for integrated chronic care provision on the basis of evidence-based care standards for type 2 diabetes care [35], COPD care [36], and vascular risk management [37] was implemented in the Netherlands. Under this system, health insurers pay a single fee to one or more of the approximately 100 regional care groups that are currently in place. Care groups are legal entities in primary care, mostly owned by GPs, which deliver care and/or subcontract (other) providers to provide services. The insurers’ bundled payment contracts cover a complete package of outpatient chronic care services for a specific condition, which is informed by national care standards [38-40].

Supporting self-management is a key element of the Dutch care standards for integrated chronic care delivery. This is illustrated by the description of the role of patients in managing their disease in the standard for type 2 diabetes care [35]:

“*Following diagnosis of type 2 diabetes by the GP, medical history, lifestyle and physical fitness are mapped. Subsequently, an individual risk profile, treatment goals, and a treatment plan are drafted based on guidelines. The treatment plan is discussed with the patient and general target values are translated into individual goals, with the patient’s contribution playing a central role. In order to allow the patient to contribute to treatment, an educational course is completed. The individual treatment plan contains targets for weight, glucose regulation, blood pressure, lipids and kidney function. Moreover, agreements are made regarding lifestyle changes, cardiovascular risk profile, feet, eyes, and kidney function. Check-ups occur at least three-monthly, paying specific attention to complaints, problems, lifestyle changes, weight, glucose regulation, blood pressure and other conditions (un)related to diabetes.”*

#### Support mode and content

Our interviews indicated that the Dutch approach to self-management support for chronically ill patients is individual- rather than group-based, and focuses on educating patients about their condition as well as about healthy behaviors and self-monitoring skills. According to respondents, patient education is still very much traditional in the Netherlands, with health professionals deciding what information and skills to teach, rather than allowing patients to identify their problems and providing them with techniques to make decisions and take appropriate actions:

*“We ask patients about their lifestyle. We give them advice about their lifestyle. And if that is not enough, we can refer them to a dietician or physical therapist”* (Nurse)*.*

Although collaborative care planning is emphasized in the Dutch care standard for type 2 diabetes, none of the interviewed professionals reported actually working with individual treatment plans. Insufficient information technology (IT) and counteracting financial arrangements were mentioned as barriers towards more individual care management:

*“We’re still very much in the development phase, searching for ways to support patients’ self-management. It’s not an integral part of the care process yet, nor has it been implemented in protocols or IT”* (GP)*.*

*“The current financing system focuses on measurable results and, in so doing, hampers self-management. GPs tell patients: ‘you have to visit four times per year, whether you need it or not’. That completely opposes any form of self-management”* (Manager)*.*

The latter comment illustrates the reports of the vast majority of professionals that there is structured follow-up of patients, which is motivated by reimbursement of care professionals on the basis of performance indicators stipulating, among others, that patients should be seen in general practice at least four times per year. Some respondents believed such ‘far-reaching’ standardization of care provision opposes self-management, while others indicated that regular monitoring of diabetes patients is key to achieving good health outcomes.

#### Support format and materials

Most respondents noted that while supporting self-management is an important goal of their care programmes, the operationalisation of this care component remains underdeveloped. Nationwide approaches do not (yet) exist and regional interventions are often not standardized in care groups’ diabetes care protocols, meaning that efforts can differ between practices and providers. The lack of proactive policymaking on self-management support was mentioned by some respondents as a barrier to broad dissemination of local ‘best practices’:

*“As far as self-management support goes, we’re still very much searching for ways to operationalize; we realize that it is important, but we still have a long way to go”* (Manager)*.*

Some groups reported using motivational interviewing or web-based education programmes, such as DIEP.info, to help patients in their efforts towards self-management. In most groups, however, support efforts appeared to be limited:

*“There is attention for patients’ self-care during consultations, but self-management support has not yet been institutionalized”* (Manager)*.*

In the broader community, cognitive-behavioral interventions are widely available for smoking cessation and physical exercise, among others, yet such programmes are rarely part of regional diabetes care packages, which are covered entirely by the basic social health insurance (SHI) package that is mandatory for Dutch citizens. Hence, additional payments might be necessary in order to gain access to such services.

#### Support providers and locations

The interviews with Dutch health care professionals suggested that in practice, nurses are most involved in supporting patients’ efforts to self-manage their disease “*simply because they have more time to do so*” (GP). General practice nurses usually see patients at least three times per year; during these quarterly check-ups – as well as during the annual, more elaborate visit with the GP – patients’ self-management needs and activities are assessed and education concerning diabetes self-monitoring is provided. When deemed necessary, patients may be referred to dieticians, physical therapists, other primary care-based health providers, and/or community services that can support them in improving their health-related behaviors.

## Discussion

In this paper, we reviewed self-management support approaches for patients with chronic conditions in 13 European countries. We find that, in general, self-management support remains relatively underdeveloped in Europe, though some countries appear further than others in implementing the key support components distinguished by the CCM, i.e. patient education, collaborative care planning, provision of self-management tools, and structured follow-up. This difference might be explained, in part, by facilitative factors in countries’ health system context, such as the financing context which might incentivise self-management support efforts, and/or to what can broadly be viewed as medical culture, including length of consultation [41], nature of doctor-patient communication [42], or interdisciplinary teamwork [43]. At the same time, although there are differences in the ‘what, how, who and where’ of support activities across countries, there are considerable similarities as well. Important commonalities were: (1) the role of nurses as main support givers, which research has shown to lead to better outcomes for the chronically ill [44-46]; and (2) the setting of support activities in primary care, which is widely regarded as most suitable to serve as ‘medical home’ for chronically ill patients [47]. Moreover, respondents from most countries reported on the presence of collaborative care planning and structured follow-up of patients’ self-management over time, as suggested by the CCM, although it is often unclear how (well) these activities are implemented in practice. Findings from recent international surveys of patients’ experiences with chronic care suggest that there are still substantial shortfalls in the actual level of patient engagement in terms of patient-provider communication, shared-decision making, and follow-up and support between visits [7,48].

The self-management support approaches reviewed here differ primarily in terms of mode, format, and materials. Across and within countries, patients are offered a wide variety of educational resources and services, ranging from written materials only to different combinations of individual and/or group-based education sessions, interactive websites, telephone services, and/or peer support. According to a systematic review by Barlow et al. (2002), diversity in self-management interventions is advisable because ‘no approach will meet the needs of all participants at all points in time’ [49]. With regard to content, support efforts in the 13 countries tend to focus primarily on the first two sets of activities distinguished in the Corbin and Strauss framework [28], namely medical and behavioral management, but less so on helping patients deal with the emotional consequences of chronic illness. Active involvement of patient associations in chronic care provision, which is the case in some countries, might be an important step towards better support for patients’ emotional management. The six-month evaluation of the Expert Patient Programme in England showed that lay-led education efforts can result in improvements in patients’ partnerships with doctors, their self-efficacy, self-reported energy levels, health-related quality of life, and psychological wellbeing [50]. In Austria, the added benefit of peer-support in the Therapy Aktiv programme is currently being evaluated [51].

The interviews with health care professionals in the Netherlands suggested that, despite the emphasis on the role of patients in recent chronic care policymaking, the actual degree of self-management support in practice remains limited, an observation also reported for other countries [7,8,48]. Care providers seem to recognize that engaging patients as partners in their care is key to achieving better health outcomes, yet experience difficulties in operationalizing this phenomenon in their daily working routines. Based on the barriers to patient participation perceived by our respondents, improvements seem necessary in existing IT arrangements and financial incentives to support the use of individual treatment plans. Moreover, it will be important to create a tighter connection between the field of health promotion and the health care system, for instance by including smoking cessation interventions as part of disease management programmes [31]. Broad implementation of self-management support and of a collaborative approach to chronic care more broadly will require a paradigm shift among health care professionals, who have traditionally been trained to take control of and responsibility for patients’ acute health problems [52]. Studies in the area of shared decision-making suggest that adoption of the so-called ‘empowerment paradigm’ – which acknowledges that chronically ill patients provide most of their care themselves – will require time and effort, and a supportive health system context in terms of medical education, care processes, quality measurement, and provider reimbursement [53]. There is a need for further research into barriers and facilitators to implementation to strengthen the dissemination and, with that, the impact of effective self-management support approaches for chronically ill patients within the financial and time-related constraints of daily health care practice.

An important strength of this study is the relatively large number of countries reviewed, which allowed us to provide a broad overview of approaches to self-management support in Europe. Adding an in-depth analysis of support activities in the Netherlands provided more insight into the actual implementation in practice. A limitation of our study is that, despite the use of a data template and the operationalisation of self-management support, country-specific descriptions of support approaches differed in their level of detail and thus some approaches might be relatively underrepresented in this paper. The most important weakness of the research, however, is that we were unable to include the patient perspective, as it was not possible to survey a sufficiently large sample of patients in each country within the time frame of our study. It is likely that patients’ perceptions of the (degree of) self-management support they receive will differ from that of researchers, policymakers/advisors, and health professionals. Existing work has highlighted how, from a patient’s perspective, support for self-management for those with chronic disease in Europe and elsewhere remains underdeveloped, with a 2011 survey of people with chronic conditions in 11 countries finding 20 to 60 percent to report that health care professionals do not help them make treatment plans they can carry out in daily life [54]. Moreover, 25 to 50 percent felt that their doctor did not spend sufficient time with them or explained things in a way that patients would find easy to understand. Combined with our own findings, these findings further stress the importance of future research in the area of self-management support.

## Conclusion

The findings from our 13-country study of self-management support approaches suggest that while Europe might increasingly be talking the talk of patient participation in chronic care, it appears to be far from walking the walk. Support activities are relatively underdeveloped and remain quite traditional, that is, focusing on medical and behavioral skills, with limited attention for the emotional consequences of illness. Reported barriers to implementation of self-management support include insufficient IT and counteractive financial incentives, but also a lack of (proactive policy to stimulate) adoption of the ‘empowerment paradigm’ in health care practice. There is a need for better understanding of how we can encourage both patients and health care providers to engage in productive interactions in daily chronic care practice, which can improve health and social outcomes. Involving patients as ‘experts’ and ‘peer supporters’ might be an important step towards improving emotional management support in chronic care. Future research should investigate to what extent barriers related to health system context and/or medical culture are hampering the implementation of effective self-management support theories in practice.

### Ethical approval

Ethical approval was not required for this research in any of the countries described, as respondents participating in the expert data template and provider interviews were approached in their professional function only and no sensitive personal information was collected. Confidentiality with respect to the interview data collected in the Netherlands was maintained by quoting participants without reference to their age, sex and role with regard to the study, i.e. data were anonymised.

## Competing interests

The authors declare that they have no competing interests.

## Authors’ contributions

AE participated in data acquisition, analysis and interpretation, and drafted the manuscript. EN, CK and AC were involved in the design and coordination of the study, data acquisition, analysis and interpretation, and helped to critically revise the manuscript. MB, KC, IDZ, AE, MF, AF, BF, RJ, ZSP, ASS, AS and BV participated in data acquisition, analysis and interpretation, and helped to critically revise the manuscript. All authors read and approved the final manuscript.

## Pre-publication history

The pre-publication history for this paper can be accessed here:

http://www.biomedcentral.com/1472-6963/13/117/prepub

## Supplementary Material

Additional file 1 Overview of approaches to chronic disease management or their equivalent in 13 European countries. Click here for file
