# Supplementary material for: Is Europe putting theory into practice? A qualitative study of the level of self-management support in chronic care management approaches
Source: BMC Health Serv Res. 2013 Mar 26;13:117. doi: 10.1186/1472-6963-13-117 (PMC3621080; doi:10.1186/1472-6963-13-117)
Supplement: Additional file 1 — Overview of approaches to chronic disease management or their equivalent in 13 European countries. [file 1472-6963-13-117-S1.docx]

**Additional files**

**Additional file 1 - Overview of approaches to chronic disease management or their equivalent in 13 European countries**

| **Name** | **Year imple-mented** | **Aim/general description** | **Target group** | **Principal coordinator** | **Distribution** | **Self-management support** |
| --- | --- | --- | --- | --- | --- | --- |
| **Austria** |  |  |  |  |  |  |
| Ambulatory after-care of stroke patients, Salzburg | 1989 | To facilitate access to specialised ambulatory care for stroke patients and enable timely rehabilitation and reduce costs through early discharge | Stroke | Team of therapists ('neuro-rehabilitation’ team)  (Working group for Preventive Medicine, Salzburg, AVOS) | Service principally accessible to all stroke patients across Land Salzburg; lack of therapists in remote areas reduces access | Access to team of occupational therapists, speech therapists and physiotherapists in one-to-one and group settings; social activities; information through events |
| Care coordination / Interface management Styria | 2002/03  (pilot) | To improve the continuity of care following discharge from hospital using a care coordinator | Patients in hospital | Care coordinator at the regional SHI fund | Introduced as pilot project in one locality, the approach was gradually extended across Styria; Graz model to be transferred into usual care | Involvement of patients and their carers in discharge planning and subsequent care arrangements including information and practical assistance such as arrangement of devices and services |
| KardioMobil  Home care for patients with chronic heart failure | 2004 (pilot) | To support patients with chronic heart failure to enhance disease (self-)management, reduce hospital admissions and complications, improve quality of life | Chronic heart failure | Trained nurse (AVOS) | Programme comprises five trained nurses operating across Land Salzburg | Education about the disease, instruction in self-monitoring, and in handling emergency situations by trained nurse; follow-up assessment of patient self-management competences and needs |
| Integrated stroke care Upper Austria | 2005 | To improve care for patients with stroke both in relation to acute care and at the interface to rehabilitation | Stroke | General practitioner, Regional SHI fund | Implemented across Upper Austria and involving all hospitals that provide acute stroke care, medical emergency services and 3 rehabilitation centres | Information (stroke awareness campaigns, brochures distributed in GP practices and hospitals, dedicated website, targeted lectures) |
| ‘Therapie Aktiv’ diabetes disease management programme | 2006 | To improve the quality of life and prolong life for people with chronic disease, to place patient at the centre of care, reduce hospitalisations | Diabetes type 2 | DMP physician (General practitioner /family physician) | Implemented in 6 of 9 states; 1 state operates separate programmes, one of which is to be integrated into ‘Therapie Aktiv’ | Education through group instruction; involvement in goal setting and timelines, with agreed targets signed jointly; regular follow-up |
| **Denmark** |  |  |  |  |  |  |
| SIKS project - Integrated effort for people living with chronic disease | 2005 | To support people with chronic conditions through coordinated rehabilitation | Diabetes type 2, asthma/ COPD, chronic heart failure, IHD, balance problems | Multidisciplinary team at healthcare centre / hospital (determined by severity of condition) | Initially implemented in Østerbro healthcare centre and Bispebjerg hospital in Copenhagen for period of three years, subsequent transfer into usual care; elements of the programme taken up by Copenhagen City and hospitals | Education and regular documentation of self-management needs and activities; involvement in developing individualised treatment plans and goal setting; access to physical exercise intervention; information |
| Regional disease management programmes | ongoing | An interdisciplinary, intersectoral and coordinated effort using evidence-based recommendations and coordination of and communication between all parties | Diabetes type 2, COPD (in preparation: CVD, dementia, musculoskeletal disorder) | DMP General practitioner | Early stage; DMPs for COPD and diabetes type 2 implemented in Capital Region (end 2010); DMPs for other conditions and/or in other regions are planned or being developed | Structured (disease specific and general) education; information; involvement in developing care treatment plan and goal setting including agreeing timeline and methods for evaluation of goals; regular assessment and follow-up of problems and needs |
| Integrated clinical pathways | 2008 (cancer), 2010 (heart disease) | To ensure fast and optimal treatment and management of patients with heart disease/cancer | Heart disease, cancer | Care ('pathway') coordinator (specialist nurse) | As a national programme, integrated clinical pathways will be implemented across Denmark | Not specified |
| **England** |  |  |  |  |  |  |
| Expert Patients programme (EEP) | 2001  (pilot) | To develop the confidence and motivation of patients to use their own skills and knowledge to take effective control over life with a chronic illness | Generalist and disease-specific | Patient / service user | 2006 government policy set to increase EEP places to >100,000 by 2012; EEP also available as online classes so in theory accessible to everyone with internet access | Education of patients by lay instructors aimed at strengthening competencies and skills to cope with chronic illness including development of care plans |
| Case management / Community matron | 2004 | To enable intensive, home-based case management for older people at risk of hospitalisation and other high-intensity service users | Older people at risk of hospitalisation | Specialist nurse | 2004 policy foresaw implementation of case management and appointment of 3,000 community matrons by all PCTs in 2007; there are now between 620 and 1,350 community matrons | Education provided by specialist nurse; involvement in development of care plan and goals; regular assessment and documentation of needs and activities |
| Partnerships for older people project (POPP) | 2005–2010 | To provide person-centred and integrated services for older people, encourage investment in care approaches that promote health, wellbeing and independence, to prevent/delay need for higher intensity or institutional care | Older people (>65 years) | Varied: multidisciplinary team (health and social care); social or 'hybrid' worker; volunteer organisation | POPP ran a total of 146 projects involving 522 organisations including the police and housing associations; 85% of projects secured funding beyond the pilot phase into usual care | Varied: involvement of older people in project development, operation and evaluation; peer support, including EEP; staff and volunteers acting as ‘navigators’ to helping older people through the system; follow-up; expert carer programme; |
| Integrated care pilot programme | 2009–2011 | To improve the quality of care and outcomes for patients, to enhance partnerships on care provision and to make more efficient use of scare resources | Generalist and disease-specific (eg diabetes, COPD, dementia) | Varied: GP-led care, multidisciplinary team working, nurse-led case management, skilled key worker-led care coordination | The pilot programme involves 16 primary care trusts | Varied: patient education and provision of self-management tools by senior nurses; training in self-management of medicines |
| **Estonia** |  |  |  |  |  |  |
| Quality management in primary healthcare | 2003 (comple-tion of GP system) | Chronic disease management as a concept not established but indirectly embedded in the overall structure and organisation of the healthcare system | Diabetes type 2, cardiovascular disease (chronic heart failure, IHD) | General practitioner | Quality management framework for diabetes and chronic CVD implemented across Estonia and covering all GP practices | Education provided by GP/family nurse; involvement in development of care plan; regular assessment and follow-up; additional support by home care nurse or social worker where necessary |
| Chronic disease management at the primary/secondary care interface | Various | Chronic disease management as a concept not established but indirectly embedded in the overall structure and organisation of the healthcare system | Multiple sclerosis, Parkinson's disease, schizophrenia, COPD | Specialist (centre); co-morbidities managed by GP in coordination with specialist | Implemented across Estonia as part of usual care | Education (specialist); involvement in development of care plan; regular assessment and follow-up; mentoring/peer-support through patient associations (eg Multiple sclerosis, Parkinson's disease); support at home by nurse or social worker where necessary |
| **France** |  |  |  |  |  |  |
| Health action by teams of self-employed health professionals (ASALEE) | 2004–2007 | To improve healthcare quality by delegating selected tasks to nurses | Diabetes, CVD | Trained nurse | ASALEE is a non-profit organisation which, as of 2007, brought together 41 GPs and 8 nurses in 18 GP practices | Education on disease provided by trained nurse |
| Sophia diabetes care programme | 2008 | To improve the coordination, efficiency and quality of diabetic care | Diabetes type I and 2 | General practitioner, in collaboration with nurse | Experimental phase targeted patients of 6,000 GPs (6.4% of all GPs) in 10 departments; expanded in 2010 to reach 17,500 GPs in 19 departments; aim to roll-out across France by 2013 | Advice and information on self-management of disease and health behaviour; facilitating communication with health professionals; access to dedicated programme website |
| *Health networks* | |  |  |  |  |  |
| Diabetes networks: REVESDIAB | 2001 | To improve the quality of care for people with diabetes type 2 | Diabetes type 2 | Pathway coordinator: General practitioner or nurse | REVESDIAB is based in 3 departments in the Paris region, involving, in 2007–2008 around 500 health professionals in Essonne department; Overall, in 2007, there were 72 diabetes networks, involving 14,000 health professionals | Information and education (eg diet); coaching by nurses; involvement in developing treatment plan towards a ‘formal’ agreement between patient and network; regular assessment and follow-up including patient ‘log-book’ completed with doctor consulted |
| Coordination of professional care for the Elderly (COPA) | 2006 | To better integrate service provision between health and social care; to reduce inappropriate healthcare use, including ER and hospital utilisation; to prevent long-term nursing home institutionalisation | Frail elderly (>65 years) | Specialist nurse as case manager | The network is established in one district of Paris only and in 2007 involved 79 out of 200 primary care physicians practising in the area | Involvement in developing treatment plan and goal setting |
| *Measures in the 2003–2007 Cancer Plan* | | |  |  |  |  |
| Protocol for disease communication and promotion of shared decision-making (Dispositif d’annonce) | 2004 | To improve the organisation of processes and competencies in discussing a cancer diagnosis, and promoting shared decisionmaking between professionals, patients and their carers | Cancer | Specialist | As part of the national cancer plan principally rolled out across the country within the timeframe of the 2003–2007 Cancer Plan; by 2006, only half of the funds set aside by regions had been used for this purpose and accessible to all newly diagnosed cancer patients | Access to dedicated time informing about the illness and support; involvement in decisionmaking; access to psychological and social support; regular assessment of patient needs; follow-up |
| Multi-disciplinary team meeting (RCP) | 2004 | To promote the systematic use of multidisciplinary team in the development of cancer care plan so as to improve the quality of cancer diagnosis, treatment and support | Cancer | 'Médecin référent' (frequently surgeon) | As part of the national cancer plan principally rolled out across the country within timeframe of 2003–2007 Cancer Plan and accessible to all newly diagnosed cancer patients | As implemented within dipositif d’annonce |
| Regional cancer networks | 2004 | To coordinate all relevant actors and levels of care in the management of cancer, and to guarantee the quality and equity of care across all regions | Cancer | As in RCP | As part of the national cancer plan rolled out across the country within the timeframe of the 2003–2007 Cancer Plan and accessible to all cancer patients | As implemented within dipositif d’annonce |
| Local cancer or local multi-pathology networks | 2004 | To facilitate the local management and monitoring of cancer patients through better integration of GPs into networks of cancer care | Cancer | General practitioner | As part of the national cancer plan principally rolled out across the country within the timeframe of the 2003–2007 Cancer Plan and accessible to all cancer patients | As implemented within dipositif d’annonce |
| **Germany** |  |  |  |  |  |  |
| Disease management programmes | 2003 | Organisational approach to medical care that involves the coordinated treatment and care of patients with chronic disease across providers on the basis of scientific and up-to-date evidence | Diabetes type 1, 2; IHD (+ heart failure), breast cancer, asthma/COPD | DMP physician | DMPs are offered by SHI funds across Germany; in 2010 there were ~2,000 DMPs for each condition; number of participating physicians varies, ~65% GPs act as DMP physician for diabetes type 2 (57% on IHD) | Education programme in group sessions; involvement in agreeing treatment goals; regular follow-up, with patient reminders for missed sessions; some SHI funds also offer telephone services to further support their members participating in DMPs |
| GP contracts | 2004 | To improve the coordination of care and strengthen the role of primary care in the German health system | Generalist (some contracts target over 65s) | General practitioner /family physician | By the end of 2007, 55 GP contracts had been concluded with GP participation varying among regions | Annual checkups; advice on preventive measures and information; assessment of cardiovascular risk factors (‘*arriba’*) supports shared decisionmaking on treatment options |
| Medical care centres (MVZ): Polikum Berlin | 2004 | To provide comprehensive, coordinated and interdisciplinary care | Generalist | Multidisciplinary team | There are ~1,500 MVZ (2010), with a total of 7,500 physicians (>80% as salaried employees [65,000 physicians work in solo practice; 19,500 in group practices]); Polikum employs 45–50 physicians | Education programmes (eg weight reduction, stress management, smoking cessation), and practical instruction (eg self-monitoring of insulin therapy) |
| Integrated care: Healthy Kinzigtal | 2005 | To establish more efficient and organised healthcare for the residents of the Kinzigtal area | Generalist | Care coordinator (physician / psychotherapist) | By the end of 2008, ~6,400 integrated care contracts had been concluded. However, content and scope varies widely; Healthy Kinzigtal involves 70 providers (2010) | Regular checkups and risk assessments; involvement in development of individual treatment/ prevention plans and goal setting; representation through patient advisory board and a patient ombudsman |
| Community nurses: Care assistant in family practice (VerAH) | 2005 | To support GP services in under-served areas | Generalist (typically targeting over 65s) | Practice assistant | Incorporated in selected GP models, see above | Access to trained case managers |
| **Hungary** |  |  |  |  |  |  |
| Care coordination pilot (CCP) | 1998/99–2008 | To incentivise providers to take responsibility for the spectrum of services (primary to tertiary care) for an enrolled population in a defined area | Generalist | Care organisation (CCO): (Groups of) general practitioners, policlinic or hospital | The CCP gradually expanded from 9 care coordinators in 1999 to 16 care coordinators in 2005 when 1,500 GP practices participated; established in the region of Veresegyház, the CCP was terminated in 2008 | Education by specialised nurses; involvement in developing treatment plan and goal setting; access to self-management tools; regular assessment of problems/accomplishments |
| Asthma disease management programme | 2004 | To enhance the quality of asthma care | Asthma | Specialist (asthma) nurse | The programme has evolved into a formal national network of asthma nurses, with around 850 trained asthma nurses across Hungary (2010); the number of pulmonary dispensaries is around 160 (2007) | Patient education on asthma; access to self-monitoring tools; involvement in treatment plan, goal-setting, decisionmaking; regular assessment of problems/accomplishments |
| Treatment (and financing) protocols | 2005 (cancer) | To control costs of treatment such as those for expensive drugs in the case of cancer care | Asthma/ COPD, CVD (heart failure, IHD, stroke), cancer | Varies by disease (eg general practitioner for hypertension; specialist for cancer) | As part of the main system, coverage, in principle, is 100%. In practice, the adherence to treatment protocols is rarely audited | Information material on cancer, hypertension and other CVD; self-management support by patient associations and by healthcare staff pre-discharge for hospitalised patients |
| Gluco.net | 2009 | To provide a decision-support tool to guide patients in the monitoring and analysis of their blood sugar levels | Diabetes types 1 and 2 | Internet-based self-management support tool | In principle, available to every patient with diabetes through the internet | Access to web-based software that permits automatic upload of self-monitoring data and feedback |
| Multifunctional community centres | Ongoing | To improve efficiency in the healthcare system through better quality of care at lower costs | Generalist | Community centre | Programme implementation is ongoing; it is anticipated that 50–60 centres/projects will be established | Patient education may be provided |
| Diabetes care management programme | Various | To improve the care of patients with diabetes type 2 through a range of measures, with nurse-led care at its core | Diabetes type 2 | Diabetes specialist (physician, nurse) | Extent to which programme has been implemented by specialist diabetes outpatient units is not well understood; in 2008, there were 176 specialist diabetes units, including 41 in Budapest | Education provided by a diabetes nurse; access to self-monitoring devices (glucometer); regular follow-up to routinely assess problems and accomplishments, both in person and by telephone |
| **Italy** |  |  |  |  |  |  |
| Leonardo Pilot Project, Puglia | 2004–2007 | To improve the quality and effectiveness of healthcare for those with chronic conditions and to facilitate systematic integration into the existing organisational framework set by local health agencies | Diabetes types 1 and 2, chronic heart failure, high cardiovascular risk | Specialist nurse | Total of 85 GPs in Puglia region (~2.5% of GPs practising in the region), working with some 30 care managers | Education based on the ‘eight priorities’ approach defined by Lorig; systematic assessment of patient needs (in person/ by telephone) and follow-up |
| Integration, Management and Assistance for diabetes (IGEA) | 2006 | National strategy to support the implementation of disease management for diabetes type 2 at regional level | Diabetes type 2 | Multidisciplinary team / nurse (case management) | Implementation at regional level has been a gradual process; 35% of GP practices in Piedmont participate (2009); as a government sponsored programme involvement of all GPs anticipated | Structured diabetes education by trained staff (specialists, nurses, GPs); involvement in developing care plan; access to self-management tools; routine assessments of problems and accomplishments |
| Project Raffaello, Marche and Abruzzi | 2007 | To assess the effectiveness of an innovative model of patient care for the prevention of cardiovascular disease on the basis of disease and care management in general practice | Diabetes types 1 and 2, cardiovascular risk | Specialist nurse | The research project involves 16 clusters of GPs participating in the experimental arm of the study | Participation in devising care plan and decisionmaking; access to coaching and follow-up activities by telephone, doctor’s office or patient’s home; access to information material on disease, services availability and lifestyle |
| From On-Demand to Proactive Primary Care, Tuscany | 2009 | A three-year strategy towards the development of a new organisational approach to healthcare that emphasises proactive patient care | Hypertension, diabetes, chronic heart failure, COPD, stroke | Multiprofessional teams ('module') (General practitioner lead, community health doctor, specialist nurse) | Two stage-implementation: initial phase in 2010 involves establishment of ~50 modules with addition of modules ongoing; project expected to go into fully operational stage in 2011 | Education and counselling; instruction in self-monitoring activities; involvement in developing and consent to care plan; regular assessments of problems and needs; support by social workers where needed |
| **Latvia** |  |  |  |  |  |  |
| General primary healthcare system | 1996/98 (PHC reform) | Not applicable | Generalist | General practitioner | Chronic disease management embedded within primary care involving all GPs | Not specified |
| **Lithuania** |  |  |  |  |  |  |
| Clinical guidelines | from 2002 | To control medication costs; to improve collaboration between primary and secondary care | Diabetes, CVD, breast cancer, chronic renal failure, multiple sclerosis, depression; high-intensity users | General practitioner /specialist (depending on condition) | Clinical guidelines should in principle be implemented across health services in Lithuania; precise data are not available | Routine assessment of clinical indicators |
| Improving intersectoral collaboration | from 2004 | To improve collaboration between health and social care | Generalist and disease-specific (diabetes, CVD, cancer, chronic renal failure, multiple sclerosis, depression, HIV/AIDS) | Nurse | Principally implemented in all 60 municipalities of Lithuania | Routine assessment of problems and accomplishments; access to psychosocial rehabilitation services in some cases (mental health) |
| **Netherlands** |  |  |  |  |  |  |
| Stroke Service Delft | 1997 (pilot) | Evolved from pilot project for improving stroke care initiated in 1997 and funded by the Netherlands Institute for Health Research and Development | Stroke | Shared care nurse | Following the experiences of pilots the government actively promoted further implementation through ‘breakthrough projects’ and benchmarking of stroke services; as a result, by 2003, each region had developed at least one stroke service (a total of 69 in 2003) | Education adapted to the wishes and needs of the individual patient and his/her carers (verbal or written; communicated in group meetings alternating with individual sessions or through media such as internet or DVD) |
| Matador disease management programme / Maastricht-Heuvelland | 2000–2006 | Builds on a pilot scheme established in 1996, which used specialised diabetes nurses to reduce the number of patients seen by medical specialists in outpatient care | Diabetes type 2 | Core team of general practitioners, specialist diabetes nurse and endocrinologist | In 2006, a total of 63 of 90 GPs (70%) in the Maastricht region participated in the Matador programme | Access to ‘Diabetes Interactive Education Programme’ (DIEP), comprising lifestyle intervention training component for providers to engage patients in the development of treatment plan and goals; DIEP website; systematic patient follow-up |
| Primary care chain for diabetes type 2 / Heuvelland | 2007 | The primary care chain for diabetes type 2 describes the whole continuum of care for diabetes patients and is financed on the basis of the bundled payment system | Diabetes type 2 | General practitioner | All regional GPs are members of RHZ Heuvelland and as such participate in the diabetes care programme (just under 90 GPs by the end of 2009); there were 97 care groups in March 2010 with bundled payment contract with a health insurer, mostly for diabetes care | Regular checkups that include education on self-management by practice nurses / specialised diabetes nurses, depending on the level of need |
| National care standard for vascular risk management | 2010 | Describes the minimum requirements for appropriate, patient-centred care along the care continuum from prevention and early detection to treatment and rehabilitation | Vascular risk | Central care giver (determined by programme) | There are relatively few care groups for the provision of vascular risk management; of 55 care groups surveyed in early 2010, two had a bundled payment contract in place for vascular risk management, 17 prepared to contract | Involvement in shared decisionmaking, development of care plan and goal setting; acquire self-management competencies through ‘task-oriented communication’; motivational interviewing; and/or ‘emotional-oriented communication’ |
| **Spain** |  |  |  |  |  |  |
| Case management, Andalucía | 2002 | To improve the quality of life of persons with chronic conditions, reduce the burden placed on carers, provide improved access to social care and rehabilitation services and reduce emergency admissions | Mental health disorders, chronic disease, the over 65s | Nurse case manager | Over a period of four to five years, more than 300 case managers, linked to primary care teams, were deployed to care for seven million residents in Andalucía | Individualised and integral assessment; case managers offer support workshops for the main carers of people included in the programme to provide information on patient care and self-care in the home; all case managers have mobile phones to be reachable by their patients |
| Expert Patients Programme, Catalonia | 2006 | To promote patient self-management; improve quality of life, knowledge, behaviour and lifestyle; involve patients in their care and increase satisfaction | Heart failure, anticoagulant therapy, and COPD | Patient / service user and primary care team | By 2010, 31 groups of EPP had been developed by 18 Primary Care teams of the Catalan Health Institute with total of 287 participants (24 as expert patients) | Education of patients by lay instructors aimed at strengthening competencies and skills to cope with chronic illness including development of care plans |
| **Switzerland** |  |  |  |  |  |  |
| Physician network Delta, Geneva | 1992 | Physician networks form part of the service structure in ambulatory care; Delta was conceived as an HMO and in 2004 transformed into a physician network | Generalist; DMPs for diabetes, heart failure and asthma under development | Primary care physicians/ General practitioners | In 2010, the Delta network comprised 160 primary care physicians (10–20% generalists, internists, GPs); in Vaud canton, the network includes 20 physicians | Regular information (two information letters per year), detailing provisions for access to health promotion and disease prevention consultations and activities; website |
| Diabaide diabetes care network | 2004 | Developed based on an inventory of the needs of diabetic patients in the region of Nyon-Morges, Canton de Vaud and the creation of a working group of healthcare stakeholders involved in diabetes care | Diabetes types 1 and 2 | Endocrinologist-diabetologist | Jointly run by the Association Réseau de Soins de la Côte (one of the five care networks operating in the canton of Vaud) and two regional hospitals | Information material; customised face-to-face self-management education and follow-up; regular assessment of problems and needs; involvement in goal setting and developing a treatment plan |
| Breast cancer clinical pathway, Lausanne University Hospital and Lausanne University | 2008–2009 | To improve the quality and efficiency of healthcare | Breast cancer | Hospital (oncology) | Currently offered by Lausanne University Hospital only but there are plans for it to be extended to other regional hospitals in the canton of Vaud | Written information; regular reassessment of the patient’s situation; shared-decisionmaking; support by trained nurses and social workers; possible access to peer support groups |
